# Graft microvascular disease in solid organ transplantation

**DOI:** 10.1007/s00109-014-1173-y

**Published:** 2014-06-01

**Authors:** Xinguo Jiang, Yon K. Sung, Wen Tian, Jin Qian, Gregg L. Semenza, Mark R. Nicolls

**Affiliations:** 1 Veterans Affairs Palo Alto Health Care System, Palo Alto, CA USA; 2 Department of Medicine, Division of Pulmonary and Critical Care Medicine, Stanford University School of Medicine, Stanford, CA USA; 3 Vascular Program, Institute for Cell Engineering, Departments of Pediatrics, Medicine, Oncology, Radiation Oncology and Biological Chemistry, and McKusick-Nathans Institute of Genetic Medicine, Johns Hopkins University School of Medicine, Baltimore, MD USA

**Keywords:** Transplantation, Graft microvascular disease, Endothelial cells, Fibrosis, Chronic rejection

## Abstract

Alloimmune inflammation damages the microvasculature of solid organ transplants during acute rejection. Although immunosuppressive drugs diminish the inflammatory response, they do not directly promote vascular repair. Repetitive microvascular injury with insufficient regeneration results in prolonged tissue hypoxia and fibrotic remodeling. While clinical studies show that a loss of the microvascular circulation precedes and may act as an initiating factor for the development of chronic rejection, preclinical studies demonstrate that improved microvascular perfusion during acute rejection delays and attenuates tissue fibrosis. Therefore, preservation of a functional microvasculature may represent an effective therapeutic strategy for preventing chronic rejection. Here, we review recent advances in our understanding of the role of the microvasculature in the long-term survival of transplanted solid organs. We also highlight microvessel-centered therapeutic strategies for prolonging the survival of solid organ transplants.

## Introduction

The microvascular circulation comprises vessels that are <150 μm and includes arterioles, capillaries, and venules [1]. Arterioles are small arteries proximal to the capillaries, and in conjunction with the terminal arteries, contribute to the majority of the resistance to blood flow. The wall of the arteriole is made up of three layers: the intima, formed by the endothelial cells (ECs) and the basement membrane, the media, made up of the internal elastic lamina apposed by one or two layers of vascular smooth muscle cells (VSMC) and the adventitia, comprises fibroblasts, collagen bundles, and nerve endings [2]. Compared with arterioles, the walls of capillaries and venules are much thinner and contain only two types of cells: ECs and pericytes. Pericytes are embedded within the endothelial basement membrane and contact ECs directly in areas where the basement membrane is absent [3]. The microcirculation provides nutrition and oxygen supply to tissues and maintains tissue hydrostatic pressure; it is essential for normal tissue function [2]. Indeed, microvascular dysfunction has been shown to be involved in a number of diseases including insulin resistance, kidney fibrosis, and systemic sclerosis [4–7]. More recently, there is an increasing appreciation that coronary microvascular dysfunction may be a cause of chest pain, indicating that the microvascular system may be a promising therapeutic target for ischemic heart diseases [8].

In solid organ transplantation, chronic allograft vasculopathy in larger vessels has long been recognized as a major limitation for the long-term survival of transplant patients [9]. However, how microvascular injury and the accompanying pathologic remodeling affects the progression of chronic rejection and graft survival is not well known. Several recent animal studies highlight the importance of the microvasculature in solid organ transplantation. In a mouse orthotopic trachea transplantation (OTT) model, our group showed that the loss of a functional microvasculature is a prominent pathology that identifies the airways that are destined to develop fibrosis [10]; in this context, ‘functional’ means that the vessels are demonstrated to be effectively transporting blood, as opposed to be only being identified histologically. We subsequently demonstrated that enhanced airway microvascular repair during acute rejection delays and attenuates chronic rejection [11]. Protection of the microvascular system from ischemia reperfusion injury (IRI) has also been demonstrated to prevent the development of chronic rejection in a rat cardiac allograft model [12]. Moreover, a number of clinical studies have shown that loss of the microvascular circulation precedes and may predispose allografts to chronic rejection or failure [13–17]. These studies suggest that a functional microvascular system is essential for the health of a solid organ transplant, and preservation of an intact microcirculation may represent a novel therapeutic strategy to prevent or attenuate chronic rejection.

The goal of this review is to provide a better understanding of the biology of the microvasculature in solid organ transplantation. We will first review the molecular and cellular mechanisms of vessel formation during development, because many of these events are recapitulated in vascular repair and regeneration in adults [18]. Next, the cycle of injury and repair seen in the transplant microvasculature will be discussed followed by a review of the mechanisms by which these microvessels can be damaged and thrombosed. The perspective will conclude with an exposition on the mechanisms employed by ECs to protect themselves from injury, the processes involved in repair of the microvasculature, and the pathways involved in pathologic remodeling and fibrosis. Based on these clinical and preclinical studies, we propose a neologism, ‘graft microvascular disease’ (GMVD) to describe microvascular abnormalities that can be observed during rejection. GMVD includes microvascular pathologies that are clearly distinct from the classical chronic graft vasculopathy, which is a diffuse concentric vascular wall narrowing that mainly affects arteries but not the microvasculature [9, 19, 20].

## Overview of developmental vessel formation and remodeling

Vasculogenesis, arteriogenesis, and angiogenesis are the major processes by which blood vessels are formed and remodeled [21]. Vasculogenesis describes the de novo emergence of primordial ECs and the vascular plexus during embryogenesis [21, 22]. It has been recognized that fibroblast growth factor 2 (FGF-2) and bone morphogenetic protein 4 (BMP4) are two essential molecules required for the specification of mesoderm and its subsequent differentiation into cells of endothelial lineage [22–26]. Vascular endothelial growth factor (VEGF) is another key regulator of embryonic vasculogenesis and acts mainly by promoting EC survival and proliferation [22]. Following its initial formation, the primitive vascular plexus is remodeled into a functional vasculature by the coordinated activation of signaling pathways induced by factors such as VEGF, retinoic acid, and transforming growth factor-beta (TGF-β) [18, 22]. Vasculogenesis was previously thought to occur only during embryogenesis. However, because of the discovery of circulating endothelial progenitor cells (EPCs) [27], which have recently been shown to promote vascular repair and improve tissue perfusion [27–29], postnatal vasculogenic activity is now considered possible.

Arteriogenesis refers to either the remodeling of an existing collateral artery/arteriole to increase its luminal diameter in response to increased blood flow or, alternatively, to a de novo process that occurs by expansion and arterialization of the capillary bed [21, 30, 31]. Smooth muscle migration, growth, and differentiation play essential roles in arteriogenesis [30]. One recent study demonstrated that macrophage prolyl hydroxylase domain (PHD) 2 haplodeficiency promoted arteriogenesis in both development and in adult mice, and that following femoral artery ligation, these mice had better perfusion. Further mechanistic studies revealed that PHD2 haplodeficiency polarized macrophages to an M2-subtype, which produced higher levels of stromal cell-derived factor-1 (SDF-1) and platelet-derived growth factor-beta polypeptide (PDGFB). This process, in turn, enhanced vascular smooth muscle cell migration and proliferation and thereby arteriogenesis [32]. Another study demonstrated that developmental and adult arteriogenesis was regulated by synectin, a widely expressed PDZ domain protein involved in intracellular signaling; this regulation occurred in an EC-autonomous manner and suggests that ECs are central to both developmental and adult arteriogenesis [33].

Angiogenesis is a process of vessel sprouting from preexisting ones [34]. Recent studies have provided tremendous insights into the fundamental aspects of vascular sprouting during development as well as in tumor angiogenesis [34–37]. In a simplified model of vascular branching, hypoxia induces the production of VEGF. VEGF then stimulates ECs to produce dynamic filopodia, which the ECs use to probe environmental cues and guide their migration; these leading cells are termed ‘tip cells’ [34]. Cells that follow the tip cells are known as ‘stalk cells’; these cells produce fewer filopodia and instead, proliferate and establish cell junctions to stabilize the new vessel sprout [35]. VEGF and Notch-induced signaling pathways are the fundamental drivers of vascular patterning and cooperate in an integrated intercellular feedback loop between the tip and stalk cells. In this signaling feedback loop, VEGF, acting through VEGFR2, induces delta-like ligand 4 (DLL4) expression in tip cells; tip cell-expressed DLL4 then activates Notch signaling in the neighboring ECs which downregulates VEGFR2 and neuropilin 1 and upregulates VEGFR1. In this manner, Notch signaling is important for promoting a stalk cell phenotype [34, 35]. The canonical Wnt/β-catenin pathway also regulates angiogenesis. This pathway promotes vascular quiescence and stability by upregulating stalk cell expression of DLL4, which subsequently activates Notch signaling in the tip cells and promotes their phenotypic switch to stalk cells [38]. In addition to the classical VEGF-Notch driven branch patterning, it was recently demonstrated that 6-phosphofructo-2-kinase/fructose-2,6-biphosphatase 3 (PFKFB3)-regulated glycolysis in ECs also plays a role in vascular sprouting by regulating the behaviors of both the tip and stalk cells [37, 39]. Notably, the principle of tip-stalk specification by Notch signaling also controls the branching frequency of tumor vessels [40, 41].

## Microvascular EC injury in transplantation

As ECs are the primary targets for alloimmune attack following transplantation [42–45], we will focus our discussion on injury to ECs of the microvasculature. We will discuss in detail the mechanisms by which immune cells, antibodies, complement factors, oxidative stress, and immunosuppressive drugs induce EC injury.

### Immune cell-mediated EC injury

In immunosuppressed patients, cytotoxic T lymphocyte (CTL)-induced EC apoptosis is the major mechanism of acute cell-mediated rejection [42, 46]. In general, CTL induces target cell apoptosis primarily through the cell-cell contact-dependent granule exocytosis of effector molecules, mainly granzyme (Gr) B, perforin, and GrA and through the death receptor, FAS/FASL, pathway [47–49]. GrB can induce target cell death through generation of an active form of BH3 interacting-domain protein (Bid), which causes increased mitochondrial permeability and subsequent release of cytochrome C and second mitochondria-derived activator of apoptosis (SMAC/Diablo). GrB can also induce cell death through release of the reactive oxygen species (ROS) from mitochondria and through direct cleavage of caspase-3 and nuclear laminin [46]. GrA, also found in CTLs, has been shown not only to directly induce target cell apoptosis [50] but also to promote monocyte production of proinflammatory cytokines such as IL-1β, TNF-α, and IL-6 [51]. These findings suggest that CTLs indirectly induce EC dysfunction or injury by increasing the production of the inflammatory mediators. Finally, while FASL induces cell apoptosis through the FAS-associated death domain protein (FADD)/caspase-8/10-mediated extrinsic pathway, it plays an uncertain role in EC death during rejection [43]. Notably, EC death attributed to alloimmunity, CTLs act predominantly through the GrB/perforin pathway, and the contribution of FAS/FASL death signaling is minimal [52]; this result might be explained by the finding that the expression level of c-FLIP, an inhibitory protein in the death pathway, is high in ECs [53]. However, ECs can be sensitized to the FAS/FASL pathway when FAS and pro-caspase 8 are induced by IFN-γ [54].

Natural killer (NK) cells use similar mechanisms as those utilized by CTLs, namely the granule and death receptor pathways, to kill target cells [55]. In addition, NK cell also kills target cells through antibody-dependent cell-mediated cytotoxicity (ADCC), which may be the primary mechanism for EC death during acute antibody-mediated rejection (AMR) [42].

Macrophages have long been known to be key cells that mediate inflammatory injury in allografts [56, 57]. Macrophages have also been shown to induce EC death in several preclinical model systems. Macrophages can induce EC apoptosis through activation of the Wnt pathway in patterning the eye vasculature during development [58]. Macrophages also induce EC apoptosis through the TRAIL signaling pathway during oxygen-induced retinopathy [59]. In addition, macrophages can also induce EC death through the production of hypochlorous acid, inducible nitric oxide synthase (iNOS)-derived NO and proinflammatory cytokines such as TNF-α [42, 60, 61]. We recently demonstrated that the lipid mediator leukotriene B_4_ (LTB_4_) produced by infiltrating macrophages in pulmonary hypertension lungs induced EC apoptosis via suppression of endothelial nitric oxide synthase (eNOS); LTB_4_ was found to induce significant EC apoptotic death in a dose-dependent manner within 24 h of culture [62]. By extension, macrophage-produced LTB_4_ may also induce allograft EC apoptosis during acute rejection. On the other hand, monocytes/macrophages have also been shown to promote angiogenesis and vascular regeneration in both transplantation and nontransplantation models [11, 63], indicating a notable plasticity in this phylogenetically ancient cell type.

Neutrophils are also found in large numbers in allografts undergoing acute rejection and are associated with graft inflammation [64, 65]. Neutrophils have been shown to contribute to allograft rejection in various preclinical models [66–68]. In the setting of organ transplantation, neutrophils are thought to injure or kill ECs through the production of ROS or degradative enzymes used to kill invading pathogens [42]. However, research from nontransplant models suggest that the neutrophil extracellular trap (NET), which are networks of extracellular fibers, primarily composed of neutrophil DNA, might be a major mechanism by which neutrophils damage the microvasculature [69]. It has been shown that following neutrophil activation by platelets or anti-neutrophil cytoplasmic antibodies (ANCAs), NET formation damages capillary ECs [70, 71]. Consistent with the finding that histones are the major mediator inducing tissue injury in sepsis [72], it was recently shown that NETs directly induce EC death, mainly by the activity of NET components such as histones and myeloperoxidase but not elastase [73]. Although no studies have examined the role of NETs in solid organ transplantation, these mechanisms may be involved in episodes of acute rejection.

### Antibody and complement-mediated EC death and proinflammatory responses

Antibody-mediated acute or chronic rejection is a pressing problem in clinical transplantation [74–79]. Both donor specific antibodies (DSA) and nondonor specific antibodies (NDSA) have been described in rejection [80, 81]. DSAs include anti-donor human leukocyte antigen (HLA) and non-HLA antibodies [82, 83] and have long been known to cause profound changes in the ECs of the allograft microvasculature [84]. Anti-donor antibodies recognize HLA class I and II antigens, as well as non-HLA antigens such as angiotensin II type I receptor, vimentin, myosin, perlecan, type IV, V, and VI collagen, MICA, MICB, and ICAM-1 [82, 85–89]. The mechanism by which NDSAs contribute to antibody-mediated rejection is thought to be through their cross-reactivity with the major HLA proteins, such as HLA-A/B/C or HLA-DR/DQ/DP, mismatches at the allele level, and polymorphic epitopes with multiple targets [76].

Alloantibodies may induce EC death by complement-dependent mechanisms [82, 90]. Full activation of the complement system and the formation of the membrane attack complex (MAC), C5b-9, directly induce cell lysis [91]. In a rat cardiac transplant model, electron microscopy revealed that MAC-induced-EC lysis was characterized by EC swelling, fragmentation, and dissolution which led to the loss or narrowing of the microvascular lumen [92]. In addition to cell lysis, MAC also induces EC apoptosis [93], through a caspase-dependent process [94]. Similarly, MAC was also shown to contribute to the destruction of the microvascular integrity in lung allografts undergoing acute rejection [95]. Our group has also demonstrated that microvascular perfusion of airway allografts was preserved when grafts were transplanted into C3-deficient recipients. Further, we showed that C3-induced microvascular injury depended on anti-donor antibodies [96]. However, while C3 deficiency generally favored the preservation of the airway microvascular circulation, it also paradoxically enhanced capillary deposition of thrombin, which led to excessive generation of C5a that caused increased vascular leakage [97]. This study illustrates how using transplant microvascular perfusion as a separate metric of therapeutic success has the possibility of revealing surprising results which might not be considered if only histology is considered. We subsequently demonstrated that inhibition of both C3 and C5 resulted in near normal microvascular perfusion during acute rejection even in the absence of T cell suppression [97]. This study is consistent with an earlier finding that showed that thrombin may act as a C3-dependent C5 convertase [98]. Other studies have demonstrated that C5a directly induced apoptosis of target cells, such as EC and adrenomedullary cells [99, 100]. Thus, it is possible that in synergy with C3 deficiency, inhibition of C5a-induced EC injury will result in enhanced microvascular protection in different forms of solid organ transplantation.

While there is tremendous evidence demonstrating that antibody-induced EC injury occurs through complement-dependent mechanisms, noncomplement-fixing anti-EC antibodies have also been identified in transplant tissue, suggesting that there are alternative mechanisms for antibody-mediated EC injury [87]. Indeed, alloantibodies can induce target cell apoptosis through the low-affinity Fc receptor for IgG, FcγRIII (CD16), on the surface of NK cells and macrophages [101]. In the last few decades, complement-independent antibody-mediated EC injury has been increasingly recognized as a relevant mechanism in allograft rejection, and this complement-independent EC injury is likely the most prominent mechanism in chronic antibody mediated rejection [101, 102].

**Fig. 1:**
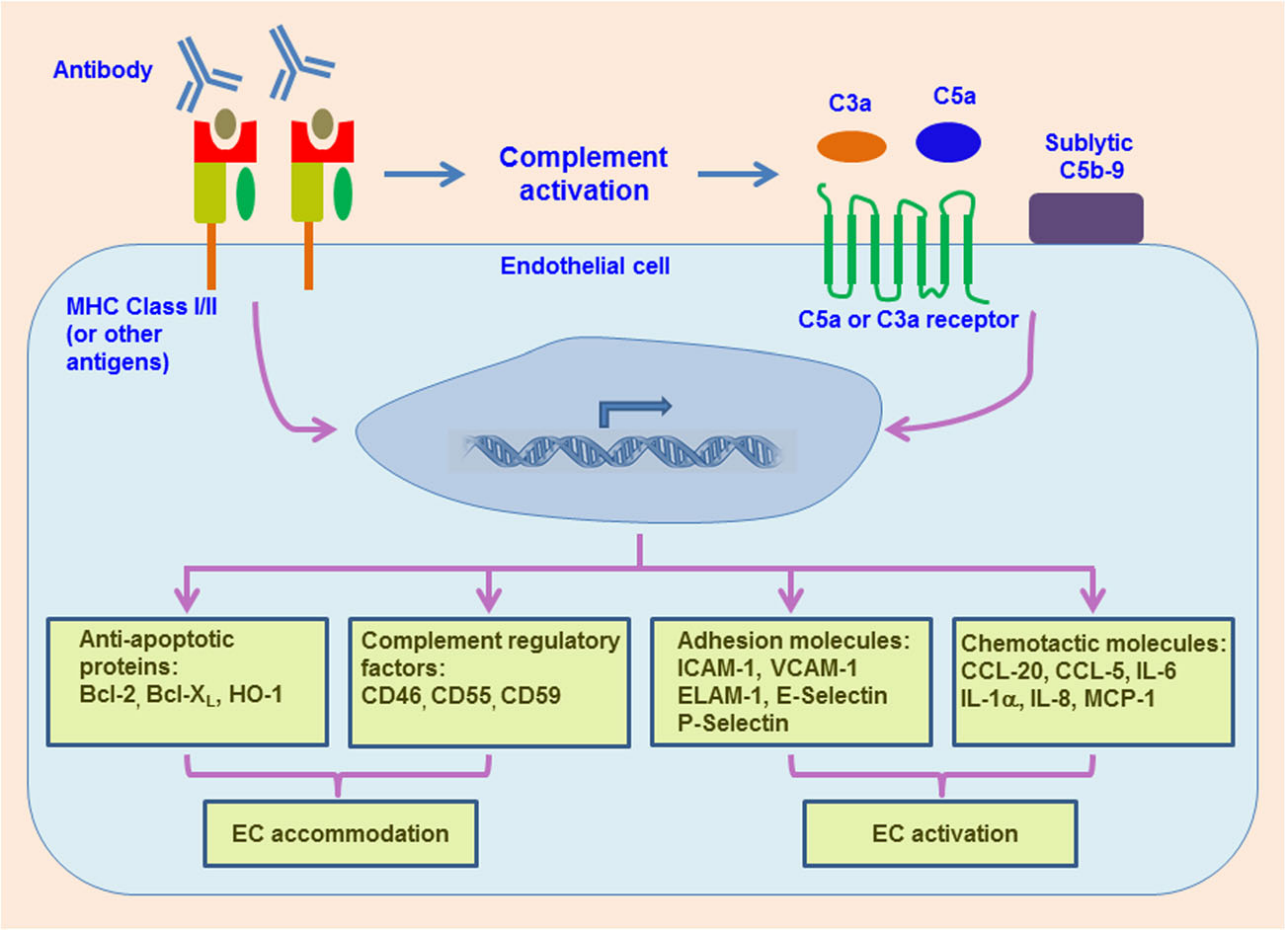
Model summarizing how antibody and complement components induce endothelial accommodation and activation. Following antibody binding to MHC molecules or binding of antibody-activated complement components, such as C3a, C5a, and sublytic concentrations of C5b-9, endothelial cells express anti-apoptotic proteins such as Bcl-2, Bcl-XL, and HO-1; complement regulatory factors such as CD46, CD55, and CD59; adhesion molecules such as ICAM-1, VCAM-1, ELAM-1, E-selectin, and P-selectin; and chemotactic molecules such as CCL-20, CCL-5, IL-6, IL-1α, IL-8, and MCP-1. EC expression of these molecules is associated with endothelial accommodation or activation. Abbreviations: *Bcl* B-cell lymphoma, *HO* heme oxygenase, *CD* cluster of differentiation, *ICAM* intercellular adhesion molecule, *VCAM* vascular cell-adhesion molecule, *ELAM* endothelial cell-leukocyte adhesion molecule, *E-selectin* endothelial cell-selectin, *P-selectin* platelet-selectin, *CCL* CC-chemokine ligand, *IL* interleukin, *MCP* monocyte chemotactic protein

EC exposure to high levels of donor-reactive antibodies usually results in its lysis or apoptosis. On the other hand, low levels of donor-reactive antibodies still lead to activation of complement, but form sublytic levels of MAC. In this situation, MAC rather than directly killing ECs leads to a proinflammatory EC phenotypic change, a process known as EC activation [43, 84] (Fig. 1). Sublytic concentrations of MAC have been shown to stimulate EC expression of the adhesion molecules, ICAM-1, VCAM-1, and ELAM-1 [103]. Complement also induces EC production of proinflammatory mediators such as IL-8, MCP-1, and IL-1α through the activation of NF-κB [104, 105], as well RANTES in an IL-1α-dependent manner [106]. In a recent landmark study by Jordan Pober’s group, a fascinating finding emerged that while alloantibody-induced MAC deposition on treated ECs, the MAC itself did not directly cause EC apoptosis but rather enhanced the recruitment of vasculopathic CD4^+^ T cells via noncanonical NF-κB signaling in ECs [107]. MAC also induces IL-6 production by vascular smooth muscle cells [108], suggesting that activated complement may also promote an inflammatory response by stimulating other types of cell layers in the microvasculature.

Anti-HLA class I antibodies can also directly activate ECs in the absence of complement by promoting Weibel–Palade body exocytosis, characterized by the release of Von Willebrand Factor (vWF) and externalization of P-selectin, a molecule that facilitates leukocyte rolling and its trafficking to the tissue parenchyma [109]. Consistent with this finding, anti-HLA class I antibodies were shown to promote macrophage recruitment into cardiac allografts, and that this was dependent on the expression of P-selectin on the EC surface [110]. On the other hand, it was recently demonstrated that complement-fixing antibodies enhanced the recruitment of monocytes compared with noncomplement-fixing antibodies through dual-activating effects on both ECs and monocytes [111]. Collectively, these studies suggest that donor-reactive antibodies can induce EC death either through complement-dependent or complement-independent mechanisms or by promoting cell-mediated immune responses.

### Oxidative stress induced EC damage

Oxidative stress can result from an imbalance between the generation and elimination of ROS and can lead to EC dysfunction or death [112]. Accumulation of excessive oxidants have been commonly seen in solid organ transplants and are attributable to a range of factors including ischemia-reperfusion injury, posttransplant graft dysfunction, use of immunosuppressive drugs as well as primary disease of the transplanted organ [113–117]. In ischemia-reperfusion injury, ROS is likely produced, initially, by donor vascular EC cells, followed by a second, much larger, burst of production by phagocytic cells such as neutrophils and macrophages [43, 118]. In lung transplants with chronic rejection, neutrophils were shown to be a major source of ROS generation [115]. The immunosuppressant, cyclosporine A, induces ROS production in hepatocytes and renal mesangial cells [119, 120]. Sirolimus also promotes ROS production by vascular cells and causes vessel dysfunction [121].

Recent studies have elucidated the mechanisms by which ROS cause EC dysfunction or death. Low concentrations of H_2_O_2_ increase EC surface expression of ICAM-1 and MHC class I molecules [122]; this finding suggests that low levels of oxidative stress do not cause irreversible injury but instead activate ECs and promote inflammation. Oxidized phospholipids also modulate the inflammatory response of ECs by inducing the unfolded protein response (UPR) [123]. Lastly, in the mouse OTT model, we have shown that ROS production is associated with apoptosis of airway microvascular ECs [124].

ROS induction of EC apoptosis may act through activation of the protein apoptosis signaling kinase 1 (ASK1) [125]. ROS may activate ASK1 by lowering intracellular levels of glutathione and reduced thioredoxin [126, 127], releasing ASK1 from its inhibitor, protein 14-3-3 [128] and activating protein kinase D (PKD), which facilitates the oligomerization and phosphorylation required for ASK1 activation [129]. Activated ASK1 then induces EC apoptosis in a JNK-dependent or JNK-independent manner [125, 130]. Oxidative stress also induced EC apoptosis through NF-κB activation [131]. These studies indicate that ECs of the transplanted organ may be subject to ROS-induced apoptosis through discrete mechanisms.

### EC damage by immunosuppressive drugs

It is now well accepted that many of the immunosuppressive drugs used to prevent rejection can cause EC damage and dysfunction [132]. Studies have shown that different types of immunosuppressive drugs induce distinct EC dysfunction. One study showed that at therapeutic concentrations, cyclosporine A, rapamycin, and mycophenolic acid all strongly induce oxidative stress in cultured human microvascular ECs and that this stimulation correlated with enhanced EC apoptosis. On the other hand, tacrolimus only slightly induced oxidative stress but led to profound increases in endothelin-1 (ET-1) production. Methylprednisolone causes the least amount of EC dysfunction [133]. Interestingly, another study showed that endothelial wound repair was significantly impaired by methylprednisolone but not by cyclosporine A and azathioprine [134]. Consistent with the in vitro findings, patients with kidney transplants treated with cyclosporine A had impaired NO production at both basal and stimulated conditions compared to patients treated with azathioprine and to healthy controls [135]. Tacrolimus also causes glomerular injury through induction of EC dysfunction by directly upregulating nicotinamide adenine dinucleotide phosphate (NADPH) oxidase activity and promoting ROS production [136]. Additionally, cyclosporine A led to microvascular endothelial dysfunction in patients with heart transplants [137]. Sirolimus (rapamycin) also causes coronary vascular dysfunction in cardiac allografts by upregulating mitochondrial superoxide release and by enhancing NADPH oxidase-driven superoxide production [121]. These preclinical and clinical studies collectively demonstrated that commonly used immunosuppressive drugs induce EC dysfunction, with excessively produced ROS as a prominent downstream effector.

## Microvascular thrombosis

The endothelium is the master regulator of microvascular thrombosis. EC expression of a number of factors is known to be prothrombotic; these factors include procoagulants, such as vWF, tissue factor (TF), thrombin receptor and PAI-1, adhesion molecules, such as ICAM-1, VCAM-1, E-selectin and P-selectin, vasoconstrictors such as ET-1 and platelet activating factor (PAF), and proapoptotic molecules such as Bax, Bad, and CCP32 [138]. Therefore, both the alloimmune response and nonimmune factor-induced EC activation or death predisposes the transplant microvasculature to thrombosis [42, 43]. In addition, immunosuppressive drugs such as cyclosporine A, tacrolimus, rapamycin, and antithymocyte globulin have all been shown to enhance thrombus formation [139]. In a clinical study, fibrin was found in the microcirculation in about 50 % of human cardiac transplants 1 month following transplantation and that fibrin deposition was associated with the development of coronary artery disease and graft failure [140]. Moreover, prothrombogenic characteristics of the microvasculature observed in the early posttransplant period in heart transplant patients were persistent in a long-term follow-up of [140, 141]. Correspondingly, a rat model of heart transplantation showed that a hypercoagulable microvasculature is associated with the development of coronary artery disease [142]. High-dose treatment with antithrombin III has been demonstrated to induce long-term survival of mouse cardiac allografts [143]. Similarly, platelet inhibition attenuated the development of fibrosis in airway allografts [144]. Thus, in addition to EC apoptosis induced by alloimmunity, microvascular thrombosis can also contribute to compromised transplant perfusion leading to chronic rejection.

## EC resistance to injury

ECs can acquire resistance to injury by upregulating a number of cytoprotective molecules. As stated above, cell-mediated EC injury depends primarily on the GrB/perforin pathway and to lesser degree, the FAS/FASL pathway. Studies from cancer biology have demonstrated that induced overexpression of proteinase inhibitor 9 (PI9), a potent endogenous inhibitor of GrB, protected cancer cells from T cell and NK cell-mediated apoptosis [145, 146]. It has also been shown that high PI9 expression in ECs protected these cells against cytolytic cell-mediated killing [147]. PI9 expression has been shown to be inducible in ECs by an NF-κB activator, phorbol ester PMA [148]. These studies suggest that EC expression of PI9 may render its resistance to cytotoxic cell-induced apoptosis.

ECs may also become resistant to antibody-mediated cell injury, a phenomenon known as accommodation [101] (Fig 1). Expression of anti-apoptotic genes such as Bcl-2, A20, Bcl-X_L_, and HO-1 has been shown to be increased in ECs of accommodated xenografts [149, 150]. Bcl-2, Bcl-X_L_, and HO-1 expression are also significantly increased in accommodated mouse cardiac transplants and silencing of Bcl-2 abolished the accommodation [151]. Increased expression of Bcl-X_L_ was found in ECs of accommodated human renal transplants with circulating anti-donor antibody [152]. This study also showed that Bcl-X_L_ expression in human ECs can be induced by exposure to low concentrations of anti-HLA antibody. Further studies demonstrated that subsaturating concentrations of anti-HLA class I antibody not only induced high expression levels of Bcl-2, Bcl-X_L_, and HO-1 but also activated the PI3K/Akt pathway, which facilitated phosphorylation and consequent inactivation of the proapoptotic molecule, Bad [153].

Complement regulation may also be involved in graft accommodation via human complement regulatory factors including CR1, decay accelerating factor (DAF, CD55), membrane cofactor protein (MCP, CD46), and CD59. Mice express complement receptor-related protein (CRRY) but not MCP. CD59 inhibits the MAC and the other factors inhibit the activation of both the classical and alternative pathways at the level of C3 convertase and C5 convertase [101]. A number of studies suggest that upregulation of complement regulatory factors plays a protective role in transplanted organs. EC expression of CD55 and CD59 has been shown to be associated with improved graft function in patients with complement deposition [154, 155]. Expression of both CD46 and CD55 is low in human lung transplants with chronic rejection [156]. Donor EC expression of CD46 in pig-to-baboon xenotransplantation is required to limit hyperacute rejection [157]. In vitro, CD55 expression can be induced by proangiogenic factors such as VEGF and FGF-2 [158]. Interestingly, VEGF-induced CD55 expression can be inhibited by cyclosporine A [159]. These studies suggest that proangiogenic factors may promote vascular repair by protecting ECs from complement-mediated injury and that immunosuppressive drugs may also cause EC injury by negatively regulating the complement regulatory factors. IFN-γ, TNF-α, and C5b-9 complex all induce EC expression of CD55, and IFN-γ with TNF-α stimulation reduces complement C3 deposition [160], suggesting a possible physiological feedback mechanism for maintaining the integrity of the microvasculature in the proinflammatory milieu of organ transplants. Nonimmune shear stress was also shown to induce CD59 expression in ECs [161] and is another mechanism by which a complement regulatory factor counteracts vaso-injurious stimuli.

## Microvascular repair

Using a functional mouse orthotopic tracheal transplant model, our group described the microvascular phenotypic change in airway transplants undergoing unmitigated alloimmune attack and the physiologic consequences of this microvascular destruction. Of note, chronic rejection developed in this model manifests mainly as subepithelial fibrosis rather than luminal fibrosis and so does not replicate the obliterative bronchiolitis (OB) lesion found in human lung transplants but is quite similar to the large airway precursor of BOS, lymphocytic bronchitis. The mechanisms associated with airway fibrosis from this model have generally been used to cautiously infer causes of fibroproliferation developing in OB lesions [10, 11]. It is possible, and perhaps likely, that more complex solid organ transplants are not revascularized in the same manner as more architecturally simple tracheas; however, use of this airway model has made it possible to divine simple ‘rules’ of vascular reorganization following rejection, rescue and remodeling. Following transplantation, the graft microvasculature in airway transplants display two general phenotypes during acute and chronic rejection respectively. In acute rejection, allografts maintain a donor-derived circulation which is undergoing both injury and concomitant repair prior to destruction. This first vascular phenotype is characterized by vessels that are relatively permeable to microspheres with evidence of the repair by donor-derived Tie2+ angiogenic cells. Transplants perfused by vessels of this phenotype can be restored to normal with immunosuppression; these allografts are never ischemic and display pseudostratified columnar epithelium without fibrosis.

The second vascular phenotype which occurs as a result of chronic rejection consists of a regrown chimeric microvasculature, largely of recipient origin, following destruction of the donor circulatory system. It is likely that, in organs with larger mass than airway allografts, that the degree of chimerism is substantially less than observed in the tracheal model. In the latter model, this vascular phenotype is characterized by new vessels that are structurally and functionally abnormal and perfuse airways now lined by flattened, cuboidal, and nonciliated epithelial cells overlying subepithelial fibrosis [11]. We think these are prototypes of GMVD. In other words, GMVD includes distinct microvascular pathologies that may appear in different rejection phases. Once the airway transplant loses its functional microvasculature, it cannot be rescued by immunosuppressive therapies and progression to chronic rejection is unrelenting [10]. Principles that emerged from this work were that just as microvessel loss following acute rejection predicted a lack of response to immunotherapy, so preventing microvessel loss could prevent chronic rejection.

The repair of donor vessels through the augmentation of endogenous cellular repair processes in both the donor and recipient may be key for maintaining a normal transplant. It is now generally accepted that the ECs which contribute to this repair process are derived both from the local vascular bed as well as from the systemic circulation [28, 162]. Because of its importance in regulating the control of angiogenesis in hypoxic tissue, we investigated the role of hypoxia inducible factor-1alpha (HIF-1α) in transplant vascular repair. We showed that HIF-1α deficiency in airway transplant donors accelerated microvascular loss, consistent with HIF-1α being an important signaling molecule in microvessel repair. We found that recipient-derived Tie2-expressing cells (i.e., cells with EC, monocyte and pericyte lineages) are present in the donor during acute rejection and that the recruitment and retention of these proangiogenic cells are regulated by donor-expressed HIF-1α and its downstream gene, SDF-1. Overexpression of HIF-1α in the donor promoted enhanced migration of recipient-derived proangiogenic cells and prolonged tissue perfusion, which in turn attenuated the development of tissue fibrosis [11]. We further demonstrated that knockdown of the VHL gene, a negative regulator of HIF, in Tie2 lineage cells of the recipient, promoted microvascular repair in the transplant [163]. This confirms that recipient-derived proangiogenic cells contribute to the repair of the donor microvasculature and provides evidence that overexpression of HIF in proangiogenic cells enhances their reparative capacity.

Together, these studies suggest that overexpression of HIF-1α in both the donor and recipient promotes allograft microvascular repair and that this enhanced repair may result from an increased expression of proangiogenic factors such as placental growth factor (PLGF), SDF-1 and to a lesser degree VEGF [11, 124, 163]. Interestingly, while EC VEGF autocrine signaling has been shown to be required for vascular homeostasis [164], excessive VEGF acting on EC in a paracrine fashion often results in immature vasculature [165]. It is therefore possible that locally overexpressed HIF-1α (especially in EC lineage cells) may promote transplant vascular homeostasis in part by inducing EC expression of VEGF, which in turn promotes its survival. Such excessive VEGF signaling may occur secondary to ‘leukocyte-induced angiogenesis,’ first described in the 1970s [166, 167]. As reviewed by Contreras and Briscoe [168], inflammation itself promotes a form of angiogenesis that is ultimately deleterious to the transplant. Early physiologic homeostatic repair of graft microvasculature in the absence of inflammation appears to be an important factor in limiting tissue fibrosis and chronic rejection. By contrast, if VEGF is delivered to the tissue, via exogenous production or by VEGF-producing leukocytes its effects may be nonphysiological and cause abnormal neoangiogenesis and disease. In the case of allograft rejection, delivery of VEGF in this manner results in a maladaptive type of angiogenesis that causes local hypoxia reminiscent of tumor neovascularization (reviewed in [169]).

While HIF-1α signaling can promote microvessel integrity, other proinflammatory pathways can foster repair, which as alluded to above may be less functional than vessels repaired in the absence of inflammation. The C5b-9 complex has also been shown to induce EC proliferation and migration in an Akt-dependent manner [170], suggesting a potential feedback mechanism for enhancing microvascular repair following alloimmune-induced inflammation. Other proinflammatory mediators produced by leukocytes may also promote EC activation, proliferation, and angiogenesis [169]. However, these newly produced vessels are abnormal and are not optimized for the delivery of oxygen and nutrition. Therefore, the ideal therapeutic strategy to promote microvascular repair should not only mitigate inflammation but also promote more physiological angiogenesis (such as vascular repair promoted by HIF-1α).

## Microvascular remodeling and fibrosis

Fibrosis is characterized by the excessive production of extracellular matrix constituents and is often a result of chronic inflammation caused by inadequate tissue repair [171, 172]. Pathological angiogenesis, also called vascular remodeling, is associated with all fibroproliferative disorders [173]. In a heterotopic mouse trachea transplantation model, CXCR2 ligand/CXCR2 signaling was associated with pathological angiogenesis and disruption of this signaling pathway attenuated late abnormal vascular remodeling [174]. Other proinflammatory mediators such as IL-1α, IL-1β and TNF-α also promote vascular remodeling [175], suggesting that pathological angiogenesis is likely promoted by the proinflammatory microenvironment of the transplanted organs.

**Fig. 2:**
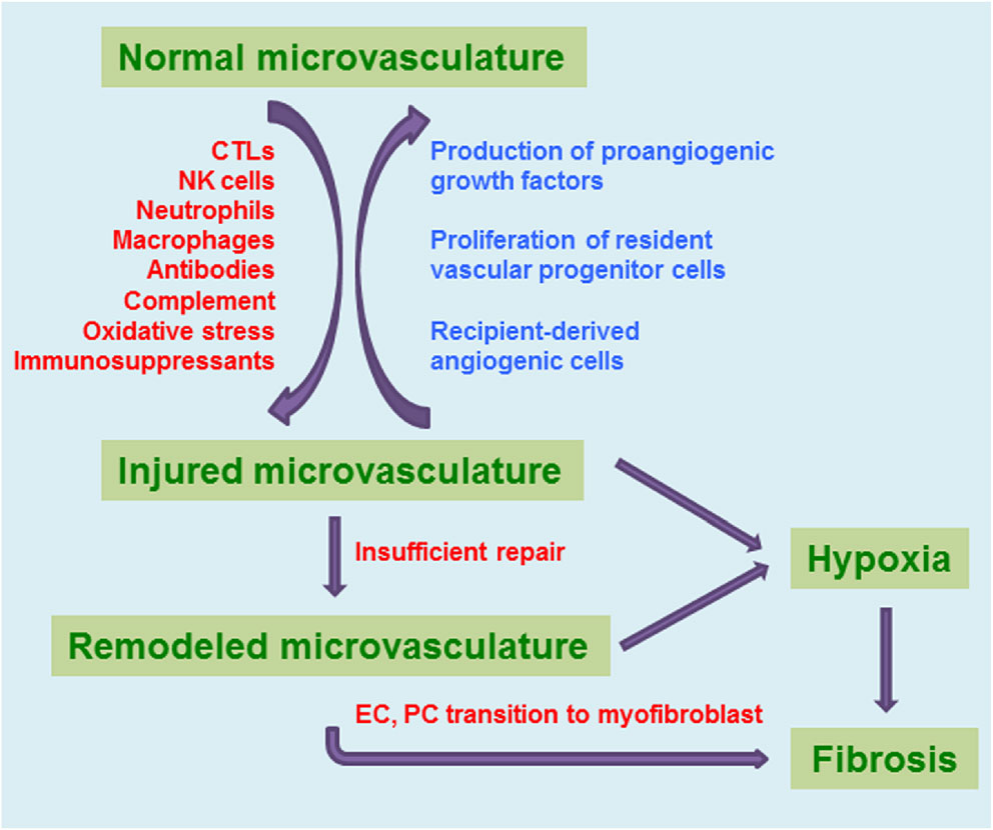
Microvascular injury and the development of fibrosis. Normal microvasculature of the solid organ transplant can be damaged by immune cells such as CTLs, NK cells, macrophages, and neutrophils; antibody, complement, oxidative stress, and immunosuppressive drugs also induce vascular injury. Damaged microvasculature can be repaired and reversed to normal through local production of angiogenic factors, proliferation of resident vascular progenitor cells, as well as recruitment of recipient-derived proangiogenic cells. Insufficient microvascular repair leads to its remodeling. Both injured and remodeled microvasculature are functionally abnormal and results in tissue hypoxia followed by tissue fibroproliferation. In addition, vascular remodeling enhances both the endothelial cell to mesenchymal and pericyte to mesenchymal transition, both of which promotes fibrosis. Abbreviations: *EC* endothelial cell, *PC* pericytes, *CTL* cytotoxic T lymphocyte, *NK* natural killer

There is an increasing appreciation that the microvasculature plays an important role in the development of fibrosis and recent studies are beginning to elucidate the mechanisms by which microvascular remodeling promotes tissue fibroproliferation [176] (Fig. 2). Hypoxia has consistently been shown to be involved in the development of lung, cardiac, liver, and kidney fibrosis [177–180]. In the mouse orthotopic tracheal transplant model, we found that microvascular remodeling starts after the loss of airway vessels. The remodeled vessels are tortuous, smaller in caliber, leaky, have sluggish blood flow, and have lower pO_2_ in the surrounding tissue, suggesting that these vessels are both structurally and functionally abnormal. Promotion of vascular repair of the airway allograft by overexpressing HIF-1α early after transplantation diminished late tissue remodeling, resulted in augmented tissue pO_2_ and is associated with a lesser degree of fibroproliferation [11, 163]. Conversely, insufficient vascular repair followed by remodeling causes prolonged tissue hypoxia which may subsequently act as a promoter of tissue fibrosis. These findings suggest that tissue hypoxia due to lack of perfusion may be a leading cause of fibrotic remodeling. Recent work has also provided ample evidence that both ECs and pericytes may differentiate into myofibroblasts and contribute to the production of extracellular matrix proteins [181, 182]. Therefore, microvascular remodeling may promote tissue fibroproliferation by multiple discrete mechanisms.

## Concluding remarks

Research over the last few decades has established that ECs are a primary target for alloimmune responses. There is also an increasing recognition that a functional microvasculature is an important determinant of the long-term health of transplanted solid organs. Given that extensive microvascular injury with insufficient repair leads to pathogenic angiogenesis and subsequent fibrosis, preservation of a healthy microvasculature by inhibiting pathways that lead to microvessel injury, increasing EC resistance to injury, or promoting vascular repair during acute rejection may represent an effective and novel therapeutic strategy for attenuating or even preventing chronic rejection. Inhibition of complement activation, oxidative stress, and thrombosis pathways may represent potential therapeutic targets for promoting microvascular health. Also, careful selection of immunosuppressive drugs is required and will be helpful in preventing unwanted EC injury. Another strategy for maintaining a healthy microvasculature is to induce EC-specific overexpression of cytoprotective molecules such as Bcl-2, Bcl-X_L_, HO-1, PI9, and complement regulatory proteins such as CD55, CD46, and CD59, all of which have been shown to promote resistance to cell- and/or antibody-mediate injury. Additionally, promotion of physiological microvascular repair such as by enhancing HIF-1α expression, especially in cells of EC lineage, during acute rejection may also be effective in preventing the development of chronic rejection; effectiveness of this approach will likely be enhanced by limiting leukocyte-driven angiogenesis (i.e., giving increased immunosuppression). Lastly, once pathological angiogenesis and accompanying fibroproliferation has started, blockade of this nonproductive vascular remodeling may also be of therapeutic efficacy. Toward this end, a better understanding of angiogenesis gained from developmental models may help to discover other effective targets for intervention.

GMVD may display distinct forms during acute and chronic rejection phases. During acute rejection, GMVD can be reversed to normal by appropriate immunosuppression with potential benefit from adjuvant therapies which promote physiological vascular repair. During chronic rejection, an emerging therapeutic goal appears to be attenuating pathological microvascular remodeling. Of note, both forms of GMVD may coexist in a transplant when different parts of the organ are in different rejection phases. Identification of the forms of GMVD within a transplant is therefore essential for optimizing new effective therapeutic interventions.
